# Overexpression of adenosine deaminase acting on RNA 1 in chordoma tissues is associated with chordoma pathogenesis by reducing miR-125a and miR-10a expression

**DOI:** 10.3892/mmr.2015.3341

**Published:** 2015-02-12

**Authors:** LEI KUANG, GUOHUA LV, BING WANG, LEI LI, YULIANG DAI, YAWEI LI

**Affiliations:** Department of Spinal Surgery, The Second Xiangya Hospital of Central South University, Changsha, Hunan 410011, P.R. China

**Keywords:** chordoma, miRNA, RNA editing, adenosine deaminase acting on RNA 1, adenosine deaminase acting on RNA 2

## Abstract

Chordoma is a rare, slow-growing primary malignant neoplasm of the axial skeleton, which arises from the remnants of the notochord. Emerging evidence suggests that microRNAs (miRs) are dysregulated in chordoma tissues and crucially involved in chordoma pathogenesis. In the present study, the expression of 11 candidate miRs were analyzed in chordoma tissues and miR-10a and miR-125a were found to be significantly downregulated compared with controls. Notably, the expression of the primary transcripts, pri-miR-125a and pri-miR-10a was unaltered, suggesting that disturbed microRNA expression may be induced by altered pri-miRNA processing. Previous studies have indicated that disturbed adenosine deaminase acting on RNA (ADAR) expression is able to alter mRNA and miRNA adenosine to inosine (A-to-I) levels associated with cancer pathogenesis. Therefore, the expression of ADAR1 and ADAR2 was analyzed in chordoma tissues. It was found that ADAR1 was significantly overexpressed, which was accompanied by enhanced pre-miR-10a and pri-miR-125a A-to-I editing. These findings suggest that ADAR2 overexpression causes enhanced pre-miR-10a and pri-miR-125a A-to-I editing, which alters mature miR-10a and miR-125a expression and may contribute to chordoma pathogenesis.

## Introduction

Chordoma is a rare tumor of the bone, known to arise from notochord remnants and is the most common primary malignant tumor of the sacrum and the mobile spine. The clinical management of chordoma is extremely challenging. Surgical resection is the primary curative strategy for chordoma, however, it is often not possible to perform adequately due to the anatomical location of the tumor (1–3). At present, there are no effective drug treatments for patients with chordoma, although radiation has been used as an adjuvant if complete resection is not possible, as well as when non-contaminated surgical margins are achieved (4,5).

A microRNA (miRNA) is a type of short, non-coding RNA that suppresses the expression of protein coding genes by partial complementary binding, particularly to the 3′-untranslated regions (3′-UTRs) of messenger RNAs (mRNAs). Alterations in the expression of miRNAs are associated with the initiation, progression and metastasis of human cancer and it is hypothesized that miRNAs function as tumor suppressors and oncogenes in cancer development (6,7). Previous studies have revealed that disturbed expression of microRNA (miR)-1 and miR-31 may be involved in chordoma pathogenesis, however, the mechanisms remain to be elucidated (8–10).

RNA editing is a widespread post-transcriptional process contributing to cellular transcriptome diversity in eukaryotes. The most well-characterized type of RNA editing identified in mammals converts cytosine to uracil and adenosine to inosine (A-to-I). In humans, the most frequent type of editing is the conversion of A-to-I, which is catalyzed by the double-stranded RNA specific adenosine deaminase acting on RNA (ADAR) family of proteins. Accumulating evidence has indicated that disturbed RNA editing may have a role in the pathogenesis of numerous types of cancers, including prostate (11), lung (12), liver (13) and malignant gliomas (14,15). Several studies have demonstrated that altered miRNA precursor editing is able to modulate miRNA processing and expression and is associated with cancer pathogenesis (15,16).

In the present study, in order to investigate the association between miRNA editing and chordoma, the sequences of miRNA precursors were compared with those of their coding regions in chordoma tissues. In addition, the expression levels of ADAR1 and ADAR1 were determined. Therefore, the present study aimed to reveal a novel association between A-to-I RNA editing and chordoma.

## Materials and methods

### Samples

Skull-base chordoma tissues and nucleus pulposus tissues were obtained from the Department of Spinal Surgery, The Second Xiangya Hospital of Central South University (Changsha, China) with approval from the ethics committee of The Second Xiangya Hospital of Central South University. Histopathological confirmation and grading was performed by pathologists at The Second Xiangya Hospital of Central South University.

### Total RNA isolation

Total RNA from eight chordoma tissues and eight nucleus pulposus tissues was isolated with TRIzol reagent according to the manufacturer’s instructions (Invitrogen Life Technologies, Carlsbad, CA, USA).

### Cell culture

Human embryonic kidney (HEK)293T cells (China Infrastructure of Cell Line Resources, Beijing, China) were cultured in Dulbecco’s modified Eagle’s medium containing 10% fetal bovine serum, 100 IU/ml penicillin and 10 mg/ml streptomycin, which were all purchased from HyClone Laboratories, Inc. (Logan, UT, USA). All cells were maintained at 37°C under an atmosphere of 5% CO_2_.

### miRNA reverse transcription-quantitative polymerase chain reaction (RT-qPCR)

RT-qPCR analysis was used to determine the relative expression level of candidate miRNAs. The expression level of miRNAs was detected by TaqMan miRNA RT-qPCR. Single-stranded cDNA was synthesized using a TaqMan MicroRNA Reverse Transcription kit (Applied Biosystems, Foster City, CA, USA) and then amplified using TaqMan Universal PCR Master mix (Applied Biosystems) together with miRNA-specific TaqMan MGB probes (Applied Biosystems). miR-RNU48, one of the most highly abundant and stably expressed miRs in human tissues, was used as an endogenous control. Each sample in each group was measured in triplicate and the experiment was repeated at least three times for the detection of miRNAs.

### DNA collection and genotyping

DNA from tissue samples was isolated using a TIANamp Genomic DNA kit (Tiangen Biotech (Beijing) Co., Ltd., Beijing, China). DNA specimens were amplified using standard PCR protocols. A total of 322 bp of the pri-miR-125a coding region and 315 bp of the pri-miR-10a coding region were obtained. The PCR products were sequenced in the forward direction using the ABI 3730xl sequencing platform (Applied Biosystems). The sequencing results were analyzed using DNAMAN 7.0 software (Lynnon Corporation, Quebec, Canada) and Chromas Lite 2.22 software (Technelysium Pty Ltd., Tewantin, QLD, Australia).

### 3′-UTR luciferase reporter assays

To generate a 3*′*-UTR luciferase reporter, the full length 3*′*-UTR from ERBB2 and homeobox A1 (HOXA1) were cloned downstream of the firefly luciferase gene into the pGL3-control vector (Promega Corporation, Madison, WI, USA). The miR-375 mimic and miR-375 inhibitor were synthesized by GenePharma Co., Ltd. (Shanghai, China). The *Renilla* luciferase-expressing plasmid pRL-TK was co-transfected for data normalization. For the luciferase reporter assays, HEK293T cells were seeded in 48 well plates. Luciferase reporter vectors were co-transfected with an miRNA mimic or miRNA inhibitor using lipofectamine 2000 (Invitrogen Life Technologies). After two days, cells were harvested and assayed with the dual-luciferase assay (Promega Corporation). Each treatment was performed in triplicate in three independent experiments. The results are expressed as relative luciferase activity (Firefly LUC/*Renilla* LUC).

### Western blotting

Protein extracts were boiled in SDS/β-mercaptoethanol sample buffer (Sigma-Aldrich, St. Louis, MO, USA) and 20 *μ*g samples were loaded into wells of 8% polyacrylamide gels. The proteins were separated by electrophoresis and the proteins in the gels were blotted onto polyvinylidene difluoride membranes (Amersham Pharmacia Biotech, St. Albans, UK) by electrophoretic transfer. The membrane was incubated with goat anti-ADAR1 polyclonal antibody (1:1,000; cat. no. sc-19077; Santa Cruz Biotechnology, Inc., Santa Cruz, CA, USA), mouse anti-GAPDH monoclonal antibody (1:5,000; cat. no. sc-365062; Santa Cruz Biotechnology, Inc.) for 1 h at 37°C. The specific protein antibody complex was detected using horseradish peroxidase-conjugated polyclonal rabbit anti-goat (1:5,000; sc-2768) or rabbit anti-mouse (1:5,000; cat. no. sc-2005) IgG (Santa Cruz Biotechnology, Inc.). Detection of the chemiluminescence reaction was performed using the ECL kit (Pierce Biotechnology, Inc., Appleton, WI, USA). The GAPDH signal was used as a loading control.

## Results

### Differential expression of miRNAs in chordoma samples

**Figure 1:**
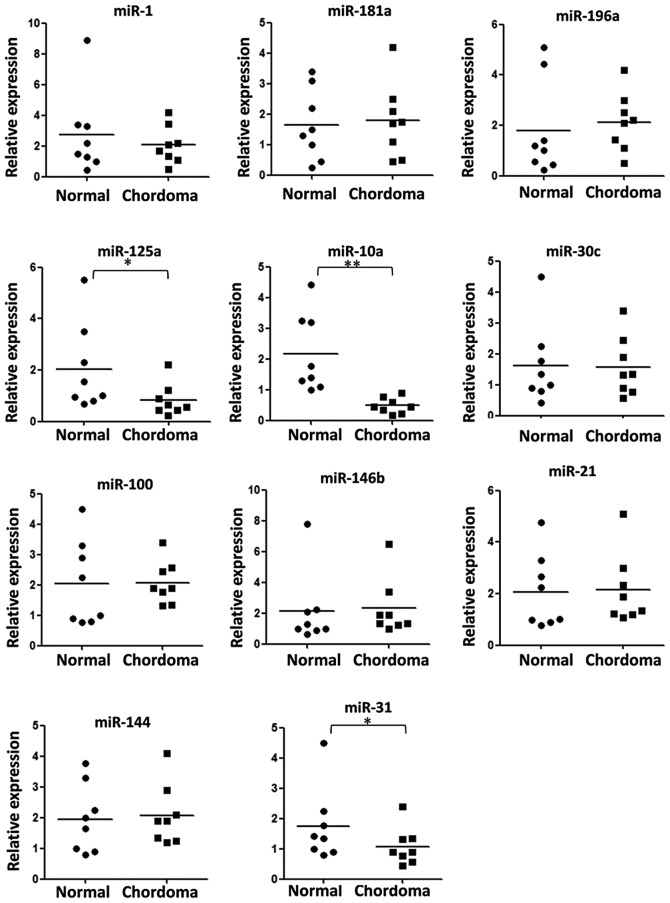
Disturbed miR expression in chordoma tissues. The expression level of 11 candidate miRs in individual samples was detected by TaqMan miRNA reverse transcription-quantitative polymerase chain reaction. Statistical analyses were performed to analyze the overall trend of each miR in all chordoma tissues. miR-RNU48 served as an internal reference among different samples and assisted in normalizing for experimental error. ^*^P<0.05 and ^**^P<0.01 vs. normal tissue. miR, microRNA.

**Figure 2:**
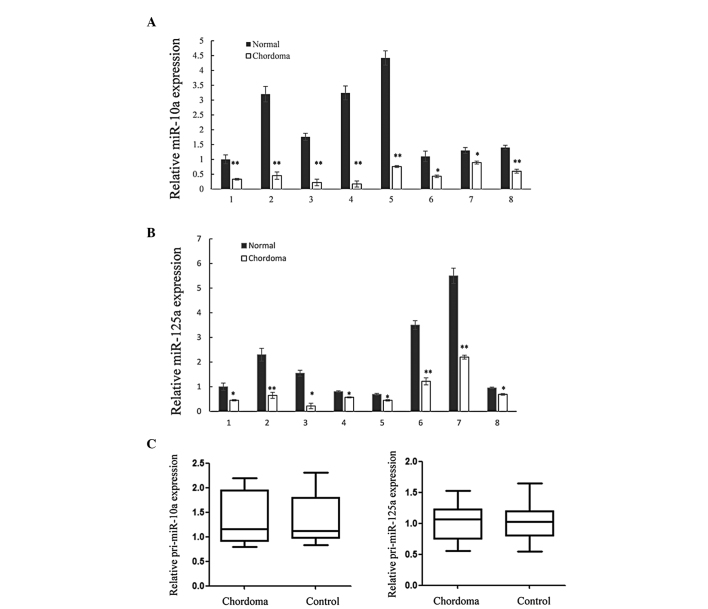
Expression of pri-miR-10a and pri-miR-125a is not altered in chordoma tissues. (A) Expression of miR-10a in chordoma and control samples. (B) Comparison of miR-125a expression in each individual sample. (C) Expression of pri-miR-10a and pri-miR-125a was detected by reverse transcription-quantitative polymerase chain reaction. Statistical analyses were performed to analyze the overall trend of miRNA precursors in all chordoma tissues. miR-RNU48 served as an internal reference among different samples and assisted in normalizing for experimental error. ^*^P<0.05 and ^**^P<0.01 vs. control. miR, microRNA.

To determine whether there are differences in the miRNA expression between chordoma and nucleus pulposus tissues, the expression levels of 11 miRNAs that are associated with cancer initiation were analyzed using RT-qPCR. The present results demonstrated that the expression of miR-10a, miR-31 and miR-125a was significantly downregulated in chordoma tissues (Fig. 1). Furthermore, only the expression of miR-10a and miR-125a was downregulated in all the sample pairs (Fig. 2). To elucidate whether the altered miRNA expression was caused at the transcriptional level or post-transcriptional level, the expression of pri-miR-10a and pri-miR-125a was also analyzed. As shown in Fig. 2C, the expression levels of pri-miR-125a and pri-miR-10a were not altered in chordoma tissues, suggesting that an altered post-transcriptional processing step was performed.

### Adenosine to guanine (A-to-G) nucleotide variations identified in sequencing results

**Figure 3:**
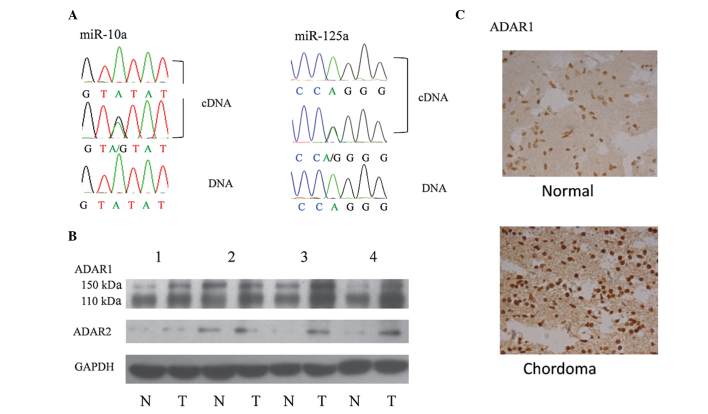
RNA editing caused by overexpression of ADAR1 and ADAR2. (A) The sequences of the pri-miR-10a and pri-miR-125a cDNA and genomic DNA were amplified and sequenced. Adenosine to guanine variation only existed in cDNA. (B) Expression of ADAR1 and ADAR2 were detected by western blot analysis. GAPDH was used as a loading control. (C) Example of ADAR1 expression level detected in a chordoma tissue and its matched non-tumor specimen. ADAR, adenosine deaminase acting on RNA; miR, microRNA.

Accumulating evidence has indicated that nucleotide mutation may disturb miRNA processing and lead to altered miRNA expression (17,18). Several studies have also reported that RNA editing is common in mammals and altered RNA editing may be associated with cancer initiation and processing (15,16). To understand the reason for the reduction in miR-10a and miR-125a expression, the DNA and cDNA sequences of these two miRNA precursors were compared. Notably, an A-to-G variation in the miR-10a and miR-125a cDNA sequence obtained from chordoma tissues was identified, which was not observed in genomic DNA and cDNA from the nucleus pulposus tissues (Fig. 3A). These results suggested that A-to-I RNA editing and overexpression of ADARs may be associated with chordoma.

### ADAR1 and ADAR2 are overexpressed in chordoma tissues

To confirm the above hypothesis, the expression of ADAR1 and ADAR2 was analyzed in chordoma and control tissues. The results of western blot analysis and immunohistochemistry indicated that the expression of ADAR1 was overexpressed in almost all the chordoma tissues with upregulated ADAR2 in a number of the cancer samples (Fig. 3B and C).

### Overexpression of ADAR1 reduces miR-10a and miR-125a expression and upregulates expression of their target genes

**Figure 4:**
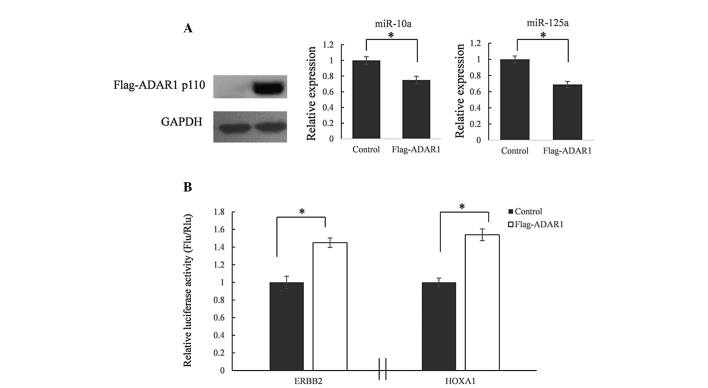
Overexpression of ADAR1 reduces miR-10a and miR-125a expression in HEK293T cells. (A) Western blot analysis was used to detect FLAG-ADAR1 expression. miR-10a and miR-125a were detected by reverse transcription-quantitative polymerase chain reaction 48 h after FLAG-ADAR1 expression vector transfection. (B) The Full length 3*′*-untranslated region from ERBB2 and HOXA1 were cloned downstream of the firefly luciferase gene into the pGL3-control vector. The PRL-TK vector containing the *Renilla* luciferase gene was used as a transfection control. The reporter vectors and FLAG-ADAR1 expression vector or empty control vector were co-transfected into HEK-293T cells. Cells were harvested and assayed using the dual-luciferase assay 48 h after transfection. Each treatment was performed in triplicate in three independent experiments. The results are expressed as relative luciferase activity (Firefly LUC/*Renilla* LUC). ^*^P<0.05 vs. control. ADAR, adenosine deaminase acting on RNA; HOXA1, homeobox A1; HEK, human embryonic kidney; miR, microRNA.

To confirm the association between overexpressed ADARs and downregulated miRNA expression, a FLAG-ADAR1 (110 kDa isoform) expression vector was constructed. In HEK293T cells, miR-10a and miR-125a were downregulated following overexpression of ADAR1 using FLAG-ADAR1 expression vector transfection (Fig. 4A).

miRNAs function as important regulators in the initiation, progression and metastasis of multiple types of cancer through suppressing gene expression (19–22). In order to determine the biological function of downregulated miRNAs, ERBB2 and HOXA1 3*′*-UTR reporter vectors were constructed, which were confirmed to be target genes of miR-10a or miR-125a. 3*′*-UTR reporter vectors and FLAG-ADAR1 expression vectors were co-transfected into HEK293T cells. After 48 h, total RNAs were extracted and miR-10a and miR-125a were detected using RT-qPCR. As shown in Fig. 4B, the relative luciferase activities were significantly increased in the ADAR1 overexpression groups.

## Discussion

Chordoma, a rare type of bone tumor originating from notochord remnants, is a slow growing malignant form of cancer. Since it is relatively rare, few molecular studies have been performed, however, further studies are required in order to enable the development of novel strategies to treat chordoma. Previous studies have demonstrated that disturbed expression of miR-1 and miR-31 may be involved in chordoma pathogenesis, however, the underlying mechanisms remain to be elucidated (8–10). In the present study, the expression of 11 miRNAs were analyzed using RT-qPCR and miR-125a, miR-10a and miR-31 were found to be significantly down-regulated. By comparing the sequences of cDNA and genomic DNA, it was identified that A-to-I RNA editing was present in the pre-miR-10a and pri-miR-125a. In addition, the expression of ADAR1 and ADAR2 was upregulated in chordoma tissues, which is collateral evidence for RNA editing.

In the present study, the expression levels of several miRNAs were detected, which have been reported tobe dysregulated in chordoma, including miR-181a, miR-31, miR-1 and miR-146b (8,9). Among them, only the reduction of miR-31 expression was in accordance with the results reported in a previous study (9). This difference may be caused by differences in ancestries and the small sample size.

It has been reported that miR-125a and miR-10a act as tumor suppressors in numerous types of cancer, including gastric cancer (23), thyroid cancer (24), lung cancer (25,26) and breast cancer (27,28). In the present study, ERBB2 (a target gene of miR-125a) and HOXA1 (a target gene of miR-10a) were selected to represent the biological function of ADAR1 overexpression. It was revealed that ADAR1 overexpression upregulated luciferase activity by reducing the expression of miR-125a and miR-10a, suggesting that ADAR1 is associated with cancer pathogenesis by reducing antitumor miRNAs.

In conclusion, to the best of our knowledge, the present study is the first to demonstrate disturbed ADAR1 expression in chordoma tissues. Overexpression of ADAR1 is associated with chordoma pathogenesis by reducing tumor suppressor miRNA expression, including miR-125a and miR-10a expression.
